# Supplementary material for: Strain-Enhanced Thermoelectric Performance in GeS2 Monolayer
Source: Materials (Basel). 2022 Jun 6;15(11):4016. doi: 10.3390/ma15114016 (PMC9182024; doi:10.3390/ma15114016)
Supplement: Supplementary file 1 [file materials-15-04016-s001.zip › materials-1751203-supplementary.pdf]

Electronic Supplementary Information for

Strain-enhanced thermoelectric performance in GeS_2 monolayer

Xinying Ruan[1,†], Rui Xiong[1,†], Zhou Cui[1], Cuilian Wen[1], Jiang-Jiang Ma[2,3], Bao-Tian Wang[2,3,4,*], and Baisheng Sa[1,*]

1 Key Laboratory of Eco-materials Advanced Technology, College of Materials Science and Engineering, Fuzhou University, Fuzhou 350100, P. R. China

2 Institute of High Energy Physics, Chinese Academy of Sciences (CAS), Beijing 100049, P. R. China

3 Spallation Neutron Source Science Center (SNSSC), Dongguan 523803, P. R. China

4 Collaborative Innovation Center of Extreme Optics, Shanxi University, Taiyuan 030006, P. R. China

* Correspondence: wangbt@ihep.ac.cn(B. Wang); bssa@fzu.edu.cn (B. Sa)

† These authors equally contributed to this work.

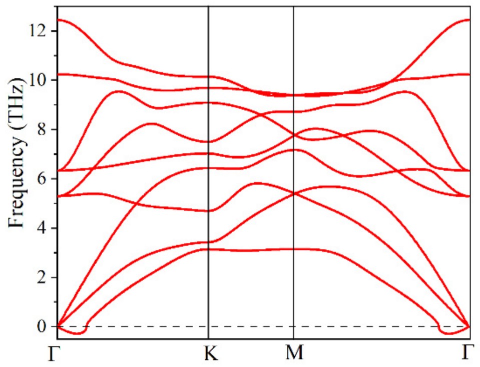

Figure S1. The phonon spectrum of GeS_2 under 2% compressive strain.

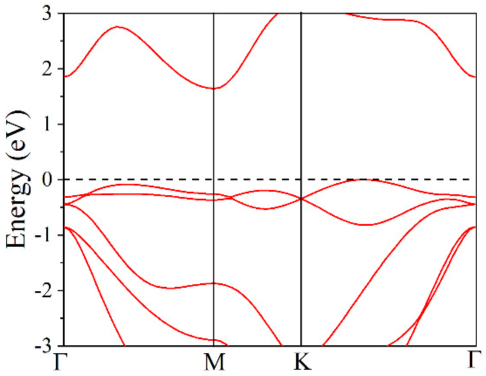

Figure S2. The band structure of GeS_2 monolayer under 8% tensile strain.

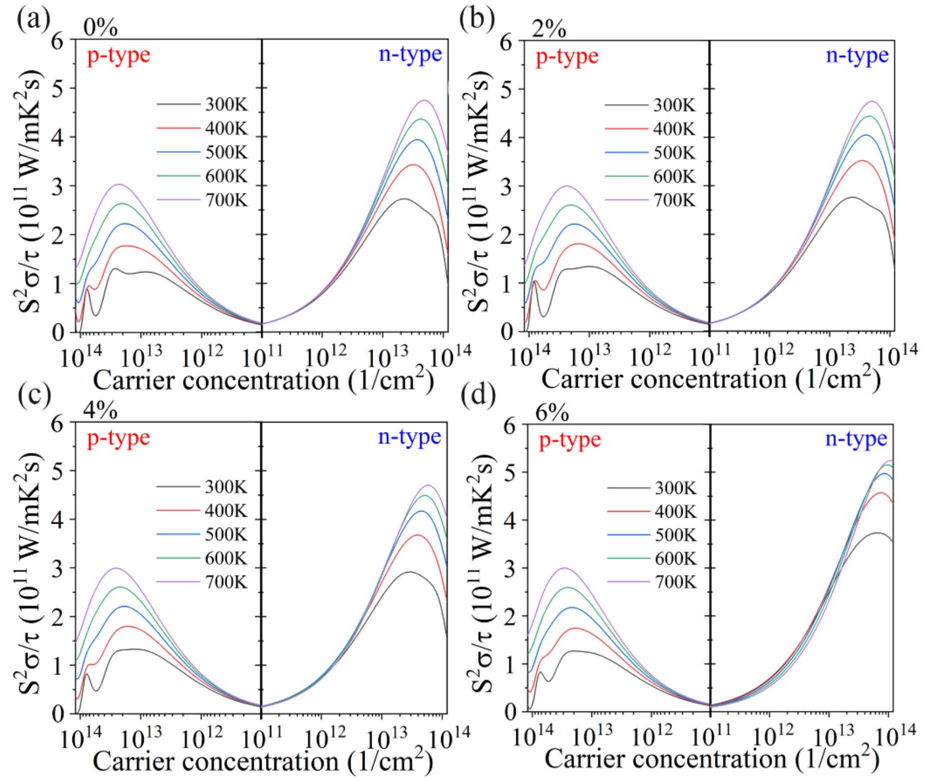

Figure S3. The power factor of GeS_2 monolayer as a function of carrier concentrations under different tensile strains.

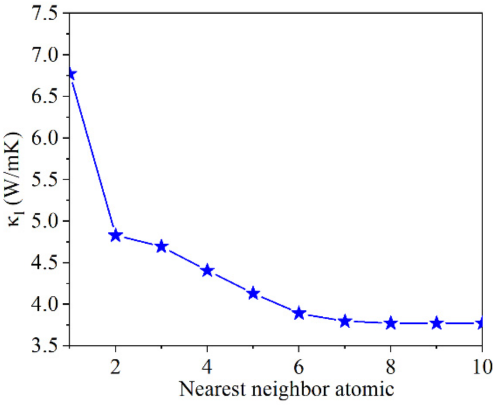

Figure S4. The lattice thermal conductivity of GeS_2 monolayer as a function of the nearest neighbor atomic.

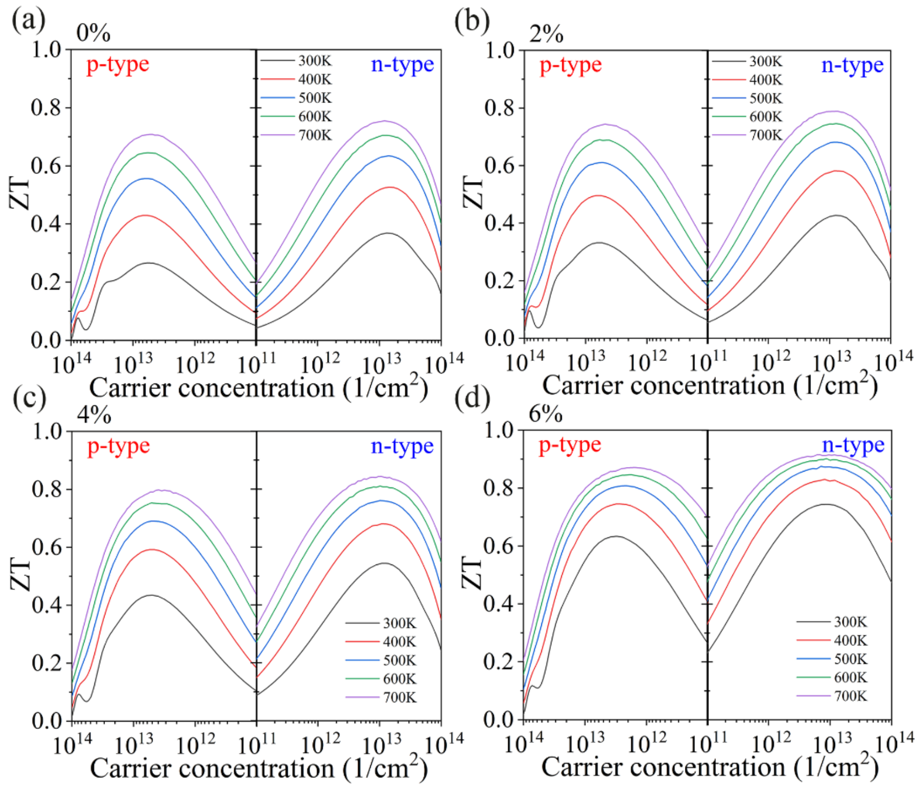

Figure S5. The figure of merit ZT of GeS_2 monolayer as a function of carrier concentrations under different tensile strains.

Table S1. The calculated parameters for effective mass of the GeS_2 monolayer, the number of band for quadratic function fitting (N_b), *k*-cutoff, band extrema points (B_p), fitting points (F_p)

Effective mass type	N_b	*k*-cutoff (1/Å)	B_p	F_p
e	27	0.015	1	6
h	26	0.015	11	6
